# Energy Consumption and Health Insurance Premiums in China's Provinces: Evidence From Asymmetric Panel Causality Test

**DOI:** 10.3389/fpubh.2021.658863

**Published:** 2021-04-29

**Authors:** Kai-Hua Wang, Lu Liu, Oana-Ramona Lobont, Moldovan Nicoleta-Claudia

**Affiliations:** ^1^School of Economics, Qingdao University, Qingdao, China; ^2^School of Management, Ocean University of China, Qingdao, China; ^3^Department of Finance, West University of Timisoara, Timisoara, Romania

**Keywords:** energy consumption, healthcare insurance premiums, asymmetric panel causality test, provincial heterogeneity, environmental pollution

## Abstract

This article employs asymmetric panel causality test to address the causal nexus between energy consumption (EC) and healthcare insurance premiums (IP) for China's different provinces. The empirical results indicate that there exist asymmetric causality runs from positive EC shocks to positive healthcare IPs in Beijing, Hebei, Tianjin, Shanxi, Inner Mongolia, Shaanxi, Gansu, Qinghai, Ningxia, Shandong, Henan, and Anhui. There is no significant link in southern and northeastern provinces mainly because of their industrial structure, high economic development level, strong insurance consciousness, and climate conditions. Therefore, governments should encourage technological innovation and further improve energy efficiency. Meanwhile, we need to optimize EC structure and raise the proportion of renewable energies. The authorities should carry out stricter environmental protection policies and protect people from pollution that comes from fossil fuel burn. The commercial health insurance should be included in the health system and become an important supplement to public health insurance.

## Introduction

In our article, we investigate the causal nexus between energy consumption (EC) and health insurance (HI) premiums (IPs) for the different provinces of China. China's EC has increased, with an annual average growth rate of 11.56%, from 1,469.64 million tons of standard coal equivalent (Mtce) in 2000 to 4,870 Mtce in 2019 (1). Because of fossil energy–dominated energy structure, China consumes a lot of coal, oil, and gas, and the proportion of renewable energies is lower than the global average level (2). The increasing EC has made China become a major emitter of pollutants (3). In 2019, China emits 9.83 billion tons of carbon dioxide (CO_2_), 4.57 million tons of sulfur dioxide (SO_2_), 12.34 million tons of nitrogen oxide (NO_x_), and 10.89 million tons of particulate matter (PM). The excessive CO_2_, SO_2_, NO_x_, PM, and other pollutants would destroy environment and cause extremely bad weather, such as severe haze and fog, which directly threat residents' health (4–6). Furthermore, the causal link between long-term exposure to pollution and morbidity, mortality, life expectancy, and hospitalization visits has been extensively discussed in scientific studies (7–10). For example, China's air pollution causes 1.6 million deaths annually, which accounts for 17% of all deaths (11). Because the problem of healthcare costs causing widespread poverty became an important issue, different public HI programs are carried out. However, the current public HI systems are insufficient to fully meet the health demands. Therefore, residents start to be aware of the bad influences and actively participate in commercial HI to cope with unexpected health problems. Commercial HI is a service that residents purchase for themselves and their relatives, which guarantees them to be taken care of when suffering unexpected health problems (12). In terms of statistics from China's National Bureau of Statistics, the IPs are only 31.6 billion RMB in 2005 and quickly increase to 706.6 billion RMB in 2019, which reaches 142% average annual growth rate. Undoubtedly, China is a fast-growing insurance market in terms of premiums in recent years, which has occupied more than 5% of global share and is expected to increase in the future. Therefore, as the largest energy consumer and a fast HI growth country, China makes us interested to discuss the potential causal link between them.

There exists a huge heterogeneity among China's different provinces, which provides another motivation for our study. China is a developing country with rapid development and own huge development imbalance among different provinces. Hence, the heterogeneity of EC may be significant for China's provinces. There is obvious disparity of EC across China's provinces in the last decade. For instance, Inner Mongolia, Hebei, Shaanxi, and some other northern provinces own heavy industry-dominated industrial structure and hence have relatively higher EC per capita. On the contrary, Guangdong, Fujian, Zhejiang, and other southern and eastern provinces depend on trade, service, and high-tech industries; hence, they do not need to consume a lot of energy in economic production and daily life. The IP per capita also demonstrates differences in China's provinces. Beijing, Shanghai, and Jiangsu are ranked as top three high levels, which correspond to high economic development level. Commonly, the Southwest provinces such as Yunnan, Guangxi, and Guizhou have low IP per capita when compared to other coastal and inland provinces. It is noticed that some provinces, such as Hebei and Shanxi, have higher IP per capita, which is inconsistent with their lower gross domestic production (GDP) per capita. These regions are big EC provinces and own fossil-dominated energy structure, which consequently lead to serious environmental problems. Bao and Zhang (13) also show that Shijiazhuang, Yuncheng, Taiyuan, Tangshan, Urumqi, Linfen, Baotou, Xianyang, Baoding, and Weinan are the top 10 cities with the poorest air quality in February 2020, and all of them are located in northern China. That means EC and related pollution may be potential reasons for IP in certain provinces. Therefore, there may exist obvious estimation biases if we investigate the causal link between EC and health IPs for China as a whole and ignore its provincial heterogeneity. With the purpose to obtain precise and convincing empirical results, our article investigates the causal nexus for China's 29 provinces, individually, which could put forward targeted policy implications for each certain province.

In this article, we find that the asymmetric causal link from EC to HI exists in Tianjin, Hebei, Shanxi, Inner Mongolia, Shaanxi, Gansu, Ningxia, Qinghai, and Anhui. That means more positive EC would stimulate residents to purchase HI to cope with unexpected events. Most of these provinces, except Anhui, are located in the north of China, which corresponds to heavy pollution regions. Our results contribute to previous studies in the following aspects. First, we examine the causal link between EC and health IP. Previous studies mainly focuses on the pollution and health concerns (14–17). In particular, air pollution is the top priority of studies (18–21). However, EC for human activities, such as manufacture, process, transport, and construction, is the basic reason in pollutant emissions (9, 22–25). Therefore, we utilize EC to replace pollution, and to the best of our knowledge, this study is the first to investigate the causal link between EC and health IPs. Second, we fully consider the heterogeneity for China's different provinces. The prior studies consider EC either in whole country (1, 26–32) or certain one province such as Beijing (3), Guangdong (33), Zhejiang (34), and Shaanxi (35). Even though these studies widely investigated EC, they did not look at the heterogeneous effects. For example, Shandong province is the largest energy consumer and consume 405.81 Mtce, which is 20 times higher than Guanxi province. As there are significant provincial heterogeneities in EC for China, the regional heterogeneities are beneficial for better understanding the causal link between EC and HI. Therefore, we employ new panel data method in our study and further clearly show causal link for different provinces, separately. Third, prior studies commonly examine causal link for whole panel data and assume symmetry in the results (36–38). However, the influences from positive and negative shocks may be different, even though the size of both shocks is exactly the same in absolute terms. As we assumed, the rising EC may increase health IPs; on the contrary, the decreasing EC may not reduce the premiums. To deal with the problem, we apply the panel asymmetric causality test proposed by Hatemi (39) to our study. The method can test the hypothesis that health IPs would produce different reaction to a positive shock than a negative shock from EC for China's different provinces. Therefore, we construct a panel system of China's 29 provinces to discuss the asymmetric causal link. Besides, the approach can deal with potential instabilities in causal link between EC and HI due to structural breaks. Finally, being different from prior studies (17, 40, 41), some important policy implications are more focused on HIHI market. We advise that the government can strengthen the propaganda of insurance consciousness to the public and offer necessary knowledge for residents to understand the insurance products. Domestic insurance companies should standardize their business, improve insurance products, and increase quality of after-sales service. Because of the low percentage of national share, foreign insurance can incorporate with local insurance and expand brand promotion, and attract customers with world-class service.

The rest of the article is constructed as follows: *Transmission Mechanism* presents the transmission mechanism between EC and health IPs. *Symmetric Panel Causality Test* shows the asymmetric panel causality test. *Data and Empirical Results* demonstrates the empirical results and discussion. *Conclusion and Policy Implications* provides the conclusion and policy implications.

## Transmission Mechanism

**Figure 1:**
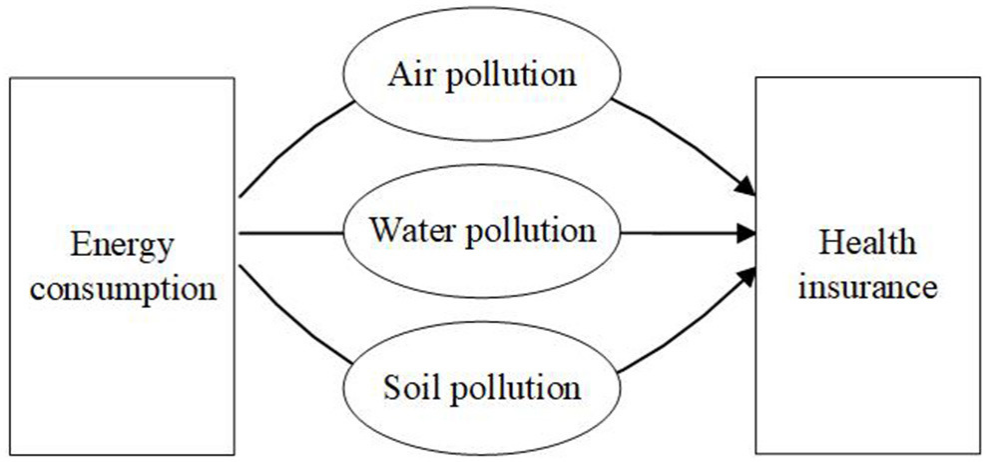
The influencing mechanism between EC and HI.

Under rapid urbanization and industrialization, China's EC is expected to keep upward trend. Figure 1 shows that the wide utilization has resulted in serious water, air, and soil pollutions, which further damage human health (10). The first long-term exposure to air pollution is easy to influence functioning of pulmonary and cardiovascular and may further cause asthma attack, heart attack, and other diseases (42). Second, there is a bidirectional relationship between EC and water resources utilization (43). For instance, water is utilized in fossil fuel–based energy production process such as coal washing, which undoubtedly results in water pollution. Conversely, the polluted water is required to be purified and discharge clean water into environment, which needs energies such as oil and coal to provide power. Third, mining activities and industrial production, relying on a large amount of EC, are regarded as main reasons for soil pollution (44). The negative impacts of air, water, and soil pollutions are not limited in natural environment, but also damage human health through food, breath, skin, and other channels. The clear evidence also proves that exposure to pollution could increase emergency department visits and hospital admission (45–47). When confronting health problems and paying for medical care, people would pursue help for HI. In China, the HI system is classified into social HI and commercial HI. The social HI is run by government to secure basic healthcare need, which has covered 95% of the population. However, the insurance faces a tight deficit that is estimated to reach 735.3 billion RMB by 2024 (48). Therefore, China starts to encourage the development of commercial HI, which is regarded as an important supplement to construct multilevel HI system. Besides, with the development of insurance companies and the richness of insurance products, the population gradually recognizes the importance of insurance and begins to purchase insurance products. To summarize, EC is basis for all kinds of economic activities that would bring a series of environmental pollutions and finally influence people behaviors for purchasing HI products.

## Asymmetric Panel Causality Test

The method is provided by Hatemi (39) and Hatemi et al. (49) that allows for asymmetric causal effect within the panel system. Thus, the positive or negative shocks may have different causal impacts in terms of this method. Presume that the focus is on testing the causal link between EC and HI in a panel model. These two variables are integrated of the first degree and can be shown as follows:

(1) $$\begin{array}{l} \text{E}{\text{C}}_{i,t}=\text{E}{\text{C}}_{i,t-1}+e_{\text{E}{\text{C}}_{i,t}}=\text{E}{\text{C}}_{i,0}+\overset{t}{\underset{j=1}{\sum }}e_{\text{E}{\text{C}}_{i,j}} \end{array}$$

(2) $$\begin{array}{l} \text{H}{\text{I}}_{i,t}=\text{H}{\text{I}}_{i,t-1}+e_{\text{H}{\text{I}}_{i,t}}=\text{H}{\text{I}}_{i,0}+\overset{t}{\underset{j=1}{\sum }}e_{\text{H}{\text{I}}_{i,j}} \end{array}$$

For *i* = 1, 2, …, *N*, where *N* shows the cross-sectional dimension. In our study, *N* is 29, which corresponds to China's 29 provinces. The positive shocks are shown as $e_{\text{E}{\text{C}}_{i,t}}^{+}=\max\left(e_{\text{E}{\text{C}}_{i,t}},0\right)$ and $e_{\text{H}{\text{I}}_{i,t}}^{+}=\max\left(e_{\text{H}{\text{I}}_{i,t}},0\right)$. The negative shocks are presented as $e_{\text{E}{\text{C}}_{i,t}}^{-}=\min\left(e_{\text{E}{\text{C}}_{i,t}},0\right)$ and $e_{HI_{i,t}}^{-}=\min\left(e_{HI_{i,t}},0\right)$. These values are utilized to obtain the cumulative sums of the positive and negative shocks as:

(3) $$\begin{array}{l} {\text{EC}}_{i,t}^{+}={\text{EC}}_{i,0}^{+}+e_{\text{E}{\text{C}}_{i,t}}^{+}=\text{E}{\text{C}}_{i,0}+\overset{t}{\underset{j=1}{\sum }}e_{\text{E}{\text{C}}_{i,j}}^{+} \end{array}$$

(4) $$\begin{array}{l} {\text{HI}}_{i,t}^{+}={\text{HI}}_{i,0}^{+}+e_{\text{H}{\text{I}}_{i,t}}^{+}=\text{H}{\text{I}}_{i,0}+\overset{t}{\underset{j=1}{\sum }}e_{\text{H}{\text{I}}_{i,j}}^{+} \end{array}$$

(5) $$\begin{array}{l} {\text{EC}}_{i,t}^{-}={\text{EC}}_{i,0}^{-}+e_{\text{E}{\text{C}}_{i,t}}^{-}=\text{E}{\text{C}}_{i,0}+\overset{t}{\underset{j=1}{\sum }}e_{\text{E}{\text{C}}_{i,j}}^{-} \end{array}$$

(6) $$\begin{array}{l} {\text{HI}}_{i,t}^{-}={\text{HI}}_{i,0}^{-}+e_{\text{H}{\text{I}}_{i,t}}^{-}=\text{H}{\text{I}}_{i,0}+\overset{t}{\underset{j=1}{\sum }}e_{\text{H}{\text{I}}_{i,j}}^{-} \end{array}$$

We next depend on vector autoregressive (VAR) model to examine the null hypothesis of no asymmetric causality within the panel system. The VAR model is effective because it takes potential dependency of the error terms across the cross-sectional units into consideration. Therefore, the asymmetric causal link between cumulative positive shocks of EC and HI are conducted in VAR model, which is shown as follow:

(7) $$\begin{array}{l} \left[\begin{array}{c} {\text{EC}}_{i,t}^{+}\\ {\text{HI}}_{i,t}^{+} \end{array}\right]=\left[\begin{array}{c} \alpha_{i0}\\ \beta_{i0} \end{array}\right]+\left[\begin{array}{cc} \overset{p}{\underset{r=1}{\sum }}\alpha_{\text{E}{\text{C}}_{i,r}} & \overset{p}{\underset{r=1}{\sum }}\alpha_{\text{H}{\text{I}}_{i,r}}\\ \overset{p}{\underset{r=1}{\sum }}\beta_{\text{E}{\text{C}}_{i,r}} & \overset{p}{\underset{r=1}{\sum }}\beta_{\text{H}{\text{I}}_{i,r}} \end{array}\right]\times \left[\begin{array}{c} {\text{EC}}_{i,t-r}^{+}\\ {\text{HI}}_{i,t-r}^{+} \end{array}\right]+\left[\begin{array}{c} \varepsilon_{\text{E}{\text{C}}_{i}}^{+}\\ \varepsilon_{\text{H}{\text{I}}_{i}}^{+} \end{array}\right] \end{array}$$

where $\varepsilon_{\text{E}{\text{C}}_{i}}^{+}$ and $\varepsilon_{\text{H}{\text{I}}_{i}}^{+}$ are the residuals. The null hypothesis of $EC_{i,t}^{+}$ does not cause ${\text{HI}}_{i,t}^{+}$, shown as:

$$\begin{array}{l} H_{0}:\beta_{EC_{i,r}}=0,\forall r \end{array}$$

where *r* = 1, 2, …, *p* and *p* is the optimal lag determined by using Hatemi-J criterion. Similarly, the negative shocks between ${\text{EC}}_{i,t}^{-}$ and ${\text{HI}}_{i,t}^{-}$ also can be tested in the same way.

The asymmetric panel causality test can recognize positive and negative shocks, respectively, and fully consider asymmetry in the results. Besides, it also allows us to take possible instabilities into the causal link, which may arise out of structural breaks. The mentioned advantages make the method widely used in many fields, including innovation expenditure (49), economic growth (50), stock market (51), and exchange rate (52). The extensive utilization proves the reliability of the method, and it can be employed in our study.

## Data and Empirical Results

Because of the missing and unavailable data in EC and/or health IP, Hong Kong, Taiwan, Macau, Xingjiang, and Tibet are not considered in our study. The sample period ranges from 1990 to 2019 for China's 29 provinces. In 1990, China's EC exceeds 1,000 Mtce for the first time and maintains fast growth rate since then. Besides, the starting point is consistent with the available time of HI statistics. According to Hao and Peng (53) and Bozoklu et al. (54), per capita EC is employed to measure EC for different provinces. Following Trinh et al. (55), per capita health IP is regarded as the main proxy for HI behaviors. This variable can better reflect the level of residents' expenditure for HI products. The data come from the China Statistical Yearbook, the China Energy Statistical Yearbook, the China Insurance Statistical Yearbook, and the provincial statistical yearbooks. Table 1 shows the descriptive statistics of EC and HI for China's different provinces. In terms of mean and median values, the EC and HI are consistent for some provinces such as Inner Mongolia, Hebei, Shanxi, Qinghai, Ningxia, and Tianjin. These provinces own heavy industry–dominated industrial structure, which emits large amount of pollution and may subsequently influence residents' HI behaviors. However, some provinces present obvious deviation for EC and HI in Guangxi, Hainan, Yunnan, and other provinces. These provinces are characterized by beautiful scenery, and tourism industry is their pillar industry, which produces little pollution and may be irrelevant to local residents' HI behaviors. Beijing, Tianjin, Shanghai, and Ningxia own positively skewed distribution, demonstrating that the tail of the right side of the distribution is fatter than the tail on the left side, and other provinces follow negatively skewed distribution. For the kurtosis, the value in EC for Shanghai is >3, indicating that its tail is fatter and obeys leptokurtic distribution. The EC and HI for other provinces submit to platykurtic distribution, demonstrating that the tails are thinner than those of a normal distribution. The unit root tests of augmented Dickey–Fuller [hereafter ADF, (56)] and Phillips–Perron [hereafter PP, (57)] are employed to examine stationarity of EC and HI for different provinces. Table 2 indicates that all variables follow unit root process at levels, but become stationary in their first difference.

**Table 1:** Summary statistics of EC and HI.

	**Mean**		**Median**		**Skewness**		**Kurtosis**	
**Province**	**EC**	**HI**	**EC**	**HI**	**EC**	**HI**	**EC**	**HI**
Beijing	3.462	8.008	3.467	7.908	0.134	0.415	1.481	2.414
Tianjin	5.093	7.022	5.264	6.941	0.511	0.739	2.177	2.616
Hebei	3.809	6.461	3.965	6.469	−0.849	−0.385	2.377	2.936
Shanxi	4.995	6.519	5.183	6.486	−0.477	−0.199	1.775	2.686
Inner Mongolia	6.825	6.151	7.194	6.078	−0.789	0.146	2.344	2.321
Liaoning	4.651	6.425	4.869	6.312	−0.831	0.162	2.558	2.343
Jilin	2.906	6.379	2.953	6.273	−0.631	0.224	2.765	2.394
Heilongjiang	2.953	6.476	3.105	6.396	−0.722	0.145	2.146	2.484
Shanghai	4.863	7.732	4.821	7.671	1.225	0.489	3.775	2.407
Jiangsu	3.856	6.902	3.568	6.889	−0.554	0.331	1.879	2.791
Zhejiang	3.232	6.597	3.282	6.589	−0.184	0.094	1.955	2.654
Anhui	1.715	6.072	1.834	6.087	−0.476	−0.221	1.698	2.976
Fujian	2.756	6.386	2.914	6.361	−0.536	−0.292	1.909	2.296
Jiangxi	1.541	5.939	1.575	5.893	−0.154	−0.024	1.667	2.289
Shandong	3.594	6.333	3.681	6.368	−0.782	0.173	2.415	2.742
Henan	2.251	6.396	2.364	6.482	−0.868	−0.584	2.402	2.789
Hubei	2.615	6.352	2.756	6.435	−0.697	−0.586	2.186	2.819
Hunan	2.184	6.047	2.269	6.075	−0.739	−0.549	2.531	3.165
Guangdong	2.615	6.656	2.693	6.607	−0.721	−0.161	2.525	2.758
Guangxi	1.743	5.515	1.889	5.558	−0.561	−0.145	1.776	2.293
Hainan	1.744	5.817	1.864	5.819	−0.263	−0.093	1.683	2.138
Chongqing	2.693	6.487	2.798	6.563	−0.678	−0.873	2.133	3.226
Sichuan	2.219	6.337	2.397	6.449	−0.771	−0.486	2.063	2.822
Guizhou	2.442	5.202	2.635	5.272	−0.589	−0.293	1.781	2.381
Yunnan	1.979	5.519	2.105	5.534	−0.518	−0.296	1.711	2.516
Shaanxi	2.561	6.378	2.714	6.405	−0.344	−0.262	1.732	2.681
Gansu	2.485	5.861	2.626	5.892	−0.409	−0.271	1.555	2.617
Qinghai	5.522	5.368	5.881	5.462	−0.228	−0.258	1.372	2.203
Ningxia	6.722	6.119	6.899	6.125	0.261	0.009	2.029	2.411

**Table 2:** The results of ADF and PP tests.

	**EC**				**HI**			
	**ADF**		**PP**		**ADF**		**PP**	
**Province**	**Level**	**First difference**	**Level**	**First difference**	**Level**	**First difference**	**Level**	**First difference**
Beijing	−0.953	−3.651^**^	−1.668	−3.807^*^	−1.649	−3.615^**^	−1.553	−3.484^**^
Tianjin	−1.909	−2.116^**^	−0.691	−2.931^*^	−1.859	−3.771^**^	−1.596	−3.809^**^
Hebei	−1.494	−2.992^*^	−1.582	−3.971^*^	−1.177	−4.678^***^	−1.425	−3.589^**^
Shanxi	−2.722	−2.916^*^	−1.853	−4.314^**^	−1.109	−2.521^*^	−1.136	−2.667^**^
Inner Mongolia	−1.624	−4.447^**^	−1.615	−5.661^***^	−1.277	−2.663^*^	−1.352	−2.107^*^
Liaoning	−1.189	−3.787^***^	−1.141	−1.818^**^	−1.699	−4.583^***^	−1.771	−3.155^**^
Jilin	−2.203	−3.812^**^	−2.493	−4.008^**^	−1.367	−3.727^**^	−1.474	−2.865^*^
Heilongjiang	−1.405	−1.925^*^	−2.711	−3.697^**^	−1.126	−3.821^**^	−1.131	−3.833^**^
Shanghai	−2.039	−4.012^**^	−2.049	−4.586^***^	−1.147	−4.355^***^	−1.187	−3.096^*^
Jiangsu	−2.726	−3.701^***^	−1.435	−3.329^**^	−1.696	−2.725^*^	−1.452	−2.799^*^
Zhejiang	−3.043	−4.335^***^	−2.398	−9.873^***^	−2.212	−3.621^**^	−1.059	−4.686^***^
Anhui	−1.734	−10.425^***^	−2.229	−3.297^**^	−1.863	−2.955^*^	−1.017	−2.754^*^
Fujian	−2.477	−2.766^*^	−1.171	−6.851^***^	−1.451	−2.815^*^	−1.474	−3.815^**^
Jiangxi	−2.608	−3.309^*^	−1.878	−3.332^**^	−1.601	−3.223^**^	−1.551	−5.483^***^
Shandong	−2.387	−2.749^*^	−2.727	−5.027^***^	−1.986	−3.924^**^	−1.716	−4.019^**^
Henan	−2.382	−3.554^**^	−1.752	−5.471^***^	−1.553	−3.625^**^	−1.576	−3.597^**^
Hubei	−1.437	−3.467^*^	−2.489	−2.769^*^	−1.492	−3.651^**^	−1.456	−3.378^**^
Hunan	−1.115	−3.111^*^	−1.776	−3.339^**^	−1.674	−2.811^*^	−1.579	−2.981^*^
Guangdong	−1.559	−3.473^**^	−2.711	−4.558^***^	−1.078	−3.363^**^	−1.112	−3.249^**^
Guangxi	−1.989	−4.063^***^	−1.925	−3.175^**^	−1.776	−3.275^**^	−1.782	−3.278^**^
Hainan	−1.283	−2.887^*^	−1.421	−2.816^*^	−1.726	−4.324^***^	−1.571	−4.277^***^
Chongqing	−1.918	−2.792^*^	−1.718	−3.242^**^	−2.131	−2.932^*^	−2.071	−3.855^**^
Sichuan	−2.303	−3.333^**^	−2.312	−3.297^**^	−1.741	−3.377^**^	−1.685	−3.326^**^
Guizhou	−1.764	−3.729^**^	−2.036	−3.629^**^	−1.897	−3.107^**^	−1.903	−3.111^**^
Yunnan	−1.609	−2.776^*^	−1.643	−3.297^**^	−1.534	−2.904^*^	−1.755	−4.257^***^
Shaanxi	−1.958	−3.326^**^	−2.464	−4.326^***^	−1.016	−3.095^*^	−1.008	−3.022^*^
Gansu	−1.493	−4.263^**^	−1.744	−3.311^**^	−1.098	−3.451^**^	−1.097	−3.416^**^
Qinghai	−1.187	−5.753^***^	−1.187	−7.745^***^	−1.898	−2.821^*^	−1.899	−2.827^*^
Ningxia	−0.977	−2.905^*^	−1.333	−4.572^***^	−1.466	−2.861^*^	−1.511	−3.261^**^

***, **, and * *denote significance at 1%, 5%, and 10% levels, respectively*.

The test for cross-sectional dependency and province-specific heterogeneity stationarity is first carried out in this study. The null hypotheses of no cross-sectional dependence and slope homogeneity are investigated by LM test [*LM*_*adj*_, (58)] and ${\tilde{\Delta }}_{adj}$ test (59), respectively. Table 3 shows that both hypotheses are rejected at the 1% significance level. The results give the following two findings. First, the shocks occur in one province would spread to the remaining 28 China's provinces. Second, we may obtain inaccurate panel causality conclusions when ignoring heterogeneity among provinces. In order to more clearly present differences among regions, seven regions are classified in our article, including North China, Northeast China, Northwest China, East China, South China, Middle China, and Southwest China. The empirical results of asymmetric causality test are demonstrated in Table 4. The symmetric Granger causality test showed that the null hypothesis that EC does not cause HI can be rejected in Gansu and Anhui at the 1% and 10% significance levels, respectively. Hence, based on the analysis for the shortcoming in symmetric Granger causality test, we further employ the asymmetric causality test to distinguish positive and negative causal links from EC to HI, respectively. Table 4 shows that the null hypothesis that positive EC does not cause positive HI can be rejected in Tianjin, Hebei, Shanxi, Inner Mongolia, Shaanxi, Gansu, Qinghai, Ningxia, Shandong, Henan, and Anhui. Meanwhile, the null hypothesis that negative EC does not cause negative HI can be rejected in none of China's 29 provinces.

**Table 3:** Cross-sectional dependence and homogeneity tests.

**Test**	**Statistics**	***p*-value**
LM_*adj*_	11.221^***^	0.002
${\tilde{△}}_{adj}$	12.333^***^	0.001

*** *denotes significance at 1% level*.

**Table 4:** The asymmetric panel causality test.

	**EC⇏HI**		**EC^+^⇏HI^+^**		**EC^−^⇏HI^−^**	
	**Wald statistics**	***p*-value**	**Wald statistics**	***p*-value**	**Wald statistics**	***p*-value**
**North China**						
Beijing	1.056	0.415	0.031	0.978	1.143	0.564
Tianjin	0.609	0.579	10.169^**^	0.043	6.319	0.253
Hebei	0.881	0.471	26.019^***^	0.008	7.437	0.119
Shanxi	0.661	0.557	17.333^**^	0.022	6.092	0.349
Inner Mongolia	0.097	0.909	44.651^***^	0.000	8.767	0.131
**Northeast China**						
Heilongjiang	2.289	0.197	1.144	0.562	0.476	0.788
Liaoning	2.816	0.152	0.993	0.609	0.572	0.751
Jilin	2.638	0.165	0.993	0.609	0.656	0.834
**Northwest China**						
Shaanxi	0.892	0.466	18.616^**^	0.037	3.384	0.771
Gansu	16.167^***^	0.007	11.779^**^	0.037	6.117	0.552
Qinghai	3.212	0.127	43.921^***^	0.003	2.368	0.871
Ningxia	1.991	0.231	9.175^*^	0.010	1.996	0.932
**East China**						
Shanghai	0.073	0.931	2.321	0.313	3.743	0.154
Jiangsu	0.875	0.479	0.751	0.687	2.374	0.305
Zhejiang	0.485	0.642	1.841	0.375	1.361	0.547
Shandong	0.343	0.725	13.262^**^	0.038	5.884	0.211
Fujian	1.656	0.281	1.774	0.183	0.152	0.697
**Middle China**						
Henan	1.065	0.412	37.908^***^	0.004	2.295	0.129
Hubei	0.881	0.471	3.144	0.132	1.335	0.248
Hunan	0.995	0.433	0.008	0.928	1.937	0.163
Anhui	3.953^*^	0.093	11.124^**^	0.045	1.349	0.509
Jiangxi	1.361	0.337	0.946	0.623	0.421	0.811
**Southwest China**						
Sichuan	0.846	0.483	0.933	0.427	4.099	0.128
Chongqing	0.032	0.969	0.945	0.623	0.421	0.817
Guizhou	0.075	0.929	0.823	0.364	0.545	0.466
Yunnan	0.636	0.568	0.097	0.755	0.039	0.843
**South China**						
Guangdong	0.466	0.652	0.822	0.364	0.185	0.677
Guangxi	0.222	0.808	0.161	0.913	1.664	0.197
Hainan	0.209	0.818	0.962	0.327	2.032	0.154

***, ** and * *denote significance at 1%, 5%, and 10% levels, respectively*.

North China includes Beijing, Tianjin, Hebei, Shanxi, and Inner Mongolia, and most provinces own the asymmetric causality from EC to HI. This region depends on coal, oil, and other fossil energies and is characterized as heavy-pollution areas. In 2018, the EC reaches 72.7, 79.73, 321.85, 201.99, and 230.68 Mtce for different provinces, which account for 1.54, 1.69, 6.82, 4.28, and 4.89% of the national total EC, respectively. At the same time, their GDP proportions are 3.6, 1.45, 3.53, 1.74, and 1.76%, respectively. We find that EC's proportions are higher than their GDP proportions in Tianjin, Hebei, Shanxi, and Inner Mongolia. These regions are also the provinces that own the asymmetric causal relationship from EC to HI. It is noticed that Hebei's EC is nearly two times its GDP proportion, which means it has low energy efficiency and produces environmental pollution. This province owns heavy air pollution and occupies 5 of the top 10 cities with the poorest air quality in 2018 (60). The serious pollution and bad environment would endanger people's health, impede economic growth, weaken authority credibility, and decrease cities' attractiveness (61). That health concerns are reflected on increasing health IPs, which reaches 222.97 billion RMB in 2019 with an annual growth rate of 165% since 2006. Northwest China, including Shanxi, Gansu, Qinghai, and Ningxia, is another region that has asymmetric causality from EC to HI. These provinces are rich in coal resources and have coal-dominated EC structure. In 2018, the proportions of coal consumption in total consumption are 72.7, 64.3, 74.1, and 85.5%, respectively. However, the national average level is 59% in the same year. That demonstrates that these provinces' coal proportions are far higher than the national level, which indicates they depend more on coal to satisfy the needs of industrial production and daily life. However, coal is a heavy pollution energy source, and its large-scale utilization is regarded as a major reason for serious air pollution in China. According to Xie et al. (62), more than 70% of PM, 85% of SO_2_, 67% of NO, and 80% of CO_2_ emissions come from coal consumption. The severe pollution has made residents to join HI for coping with unexpected diseases. The percentage of regional health IPs in total IPs has increased from 3.07 to 4.29% during the period of 2006–2018.

The distribution of above provinces is concentrated in certain regions, and some other provinces also have the asymmetric causality such as Shandong, Henan, and Anhui. Shandong and Henan have similar reasons for explaining the asymmetric causalities. First, both provinces have more than 100 million residents and ranks top three among all China's provinces, which produce huge demand for energies. In particularly, Shandong is the largest energy consumer and reaches 386.84 Mtce in 2018, which undoubtedly produce negative influence on environment. Second, both provinces own heavy industry–dominated industrial structure, which needs a large number of energies and discharge a lot of pollutants. For example, the wastewater discharge in Shandong reaches 828.58 million tons, but Jiangsu, the second largest economic province, is only 53.55 million tons. Third, both provinces are bounded by North China and Northwest China, which makes Shandong and Henan influenced by pollutants outside the provinces. Especially in winter, under the influence of the northeast monsoon, dust and haze would rapidly go south and affect the two provinces. Because of the protentional reasons, residents change their insurance behaviors and purchase HI products. In 2019, health IPs are 431.87 and 338.49 billion RMB for Shandong and Henan, which increase 135 and 275%, respectively. Apart from Shandong and Henan, Anhui is the other province that has asymmetric causal relationship. Anhui's annual average economic growth rate reaches 10.83% between the years 2000 and 2014, which is higher than other provinces and makes its EC range from 70.69 to 130.52 Mtce. The increasing EC results in ecosystem disorders and environmental pollution (63). Even though Anhui is located in South of China, it often suffers from sandstorm, acid rain, and other disastrous weather. Those negative influences from environmental pollution make residents start to pay attention to their own health. The health IP reaches 152.33 billion RMB in 2019, which increases 208% compared with that in 2007.

Other provinces do not have the symmetric and asymmetric causality between EC and health IPs, which may be shaded by some potential reasons. Northeast China, including Heilongjiang, Jilin, and Liaoning, used to be one of the four major economic regions, once plays a key role in the industrialization process. However, the region suffers from persistent negative impacts of past planned economy system and is far behind the eastern coastal provinces (64). We find that the GDP growth rates for these provinces in 2019 are 5.5, 4.2, and 3%, respectively, which rank at the bottom of all provinces in China. Accompanied by low economic development, their ECs also present an inverted U shape and are in the downward trend. The percentage of regional health IPs in all provinces is 7.45% 2019, which is almost the same as it is a decade ago. That means the weak economy is the potential reason for explaining the non-causality between EC and HI for this region. For East China, except for Shandong, other provinces containing Shanghai, Jiangsu, Zhejiang, and Fujian do not have asymmetric causality. This region is one of the regions with most vitality, and their GDPs per capita far exceed the national level. We find that health IPs per capita in Shanghai, Jiangsu, and Zhejiang are 3,459.88 RMB, 2,748.42 RMB, 2,000.52 RMB, which ranks second, third, and fourth places among all provinces. The high level of economic development makes residents have insurance consciousness early and less influenced by environmental pollution. On the contrary, their ECs per capita are 4.904, 3.914, 3.718, and 3.296, respectively, and lower than Tianjin, Inner Mongolia, and other provinces. That means these provinces have more efficient energy utilization, which emits less pollutants and reduces negative impacts on environment (1, 34). For South China and South East China, these regions are characterized by their high-quality environment (65–67). Hainan, Sichuan, Yunnan, Guizhou, Guangxi, Chongqing, and Guangdong have subtropical monsoon climate, which could bring abundant rainfall and increased environmental quality. More importantly, being different from the thermal power in North China, these provinces depend more on hydropower and nuclear power, which produce almost no pollutants (68–72). The finite climate and clean energy structure produce little impacts on local environment, and hence residents' health IPs are less influenced.

In summary, we find that the causal link between positive EC and positive HI exists in some northern provinces except Anhui. That can be attributed to coal-dominated energy structure, heavy industry–dominated industrial structure, and dry climate conditions. On the contrary, southern provinces own superior conditions in the above aspects, which makes their insurance behaviors less influenced by EC. Meanwhile, no causal relation is found for negative EC and negative HI. That indicates that once people get into the insurance habit, EC or environmental pollution cannot easily change their behaviors.

## Conclusion and Policy Implications

This article employs the asymmetric panel causality test to investigate the causal link between EC and health IPs for China's different provinces. Being different from traditional symmetrical Granger causality, our method could distinguish positive and negative shocks from EC and run Granger causality test with health IPs, respectively. The empirical conclusions show that the positive shocks in EC would result in health IP increase in Tianjin, Hebei, Shanxi, Inner Mongolia, Shaanxi, Gansu, Qinghai, Ningxia, Shandong, Henan, and Anhui. Meanwhile, Beijing, Shanghai, and other provinces do not have the causal link, which can be attributed to the political status, high energy efficiency, high GDP per capita, good insurance consciousness, and better climate conditions. China is now the largest energy consumer in the world and is expected to still maintain stable growth. That means the emission of pollutants will also increase and produce more pressure on environment, which would damage residents' health.

Hence, we provide policy implications in the following aspects. First, the authorities ought to strengthen environmental supervision and firmly enforce the law. For instance, environmental laws need to be improved to deal with new problems that appear in current pollution prevention and control. The government can utilize commercial ways, include trade carbon emissions rights, levy pollution taxes, and increase the pollution penalty to reduce pollution behaviors. Meanwhile, fiscal subsidies can also be provided to help enterprises update equipment and adopt advanced production technologies. Second, we need to persist in optimizing EC structure and increase the proportion of renewable energies. The wide utilization of coal, oil, and gas will lead to heavy environmental pollution. Hence, combining comprehensively utilized orientation and market regulation mechanisms is an effective way to transfer current EC structure and promote low-carbon industrial process. Besides, the social capital is encouraged to enter energy sector, especially clean energy, which is beneficial for increasing market vitality. Third, the HI system, including public and commercial insurance, should be improved. The government can take health compensation for provinces with bad environmental quality into consideration, which can meet residents' basic medical needs. More importantly, commercial insurance should play a more important role in the health system. Insurance companies can design insurance products for certain regions. For example, Hebei is seriously influenced by air pollution, and the insurance companies can design air pollution–related products, which may attract more residents to insure. That not only can provide more choices for people, but also increase IP income. Last, we need to introduce a competing mechanism. China has passed the Foreign Insurance Company Regulatory Law in 2002 and allowed joint ventures of up to 50% foreign ownership. However, their share of private HI market is still very limited. Therefore, we can further relax the restrictions and foster competitive environment between foreign insurance companies and local ones.

## Data Availability Statement

Publicly available datasets were analyzed in this study. This data can be found here: www.wind.com.cn.

## Author Contributions

K-HW: conceptualization, methodology, and software. O-RL: data curation and writing—original draft preparation. MN-C: visualization and investigation. All authors contributed to the article and approved the submitted version.

## Conflict of Interest

The authors declare that the research was conducted in the absence of any commercial or financial relationships that could be construed as a potential conflict of interest.
